# Reflections on the Brain’s Energy Crisis During Hypoglycemia

**DOI:** 10.3390/cimb48101016

**Published:** 2026-10-01

**Authors:** Peter Schönfeld

**Affiliations:** Institute of Biochemistry and Cell Biology, Faculty of Medicine, Otto von Guericke University Magdeburg, 39120 Magdeburg, Germany; peter.schoenfeld@med.ovgu.de

**Keywords:** hypoglycemia, brain, energy metabolism, glucose, lactate, fatty acids

## Abstract

Hypoglycemic metabolism in humans is caused by prolonged starvation, the interruption of the newborn’s body metabolism upon separation from maternal circulation, or insulin-induced acute glucose shortage. However, only acute hypoglycemia has the potential to trigger a severe energy crisis in the brain. Signal transmission by pre- and postsynaptic nerve terminals makes the greatest contribution to the brain’s energy needs. Moreover, the brain’s cognitive function depends on the transmission of multiple signals, and consequently, it is tightly connected with the energy homeostasis in brain tissue. Nevertheless, the energy crisis during acute hypoglycemia is surprising because the brain theoretically has the ability to fuel its energy metabolism using lactate and fatty acids from the circulation. However, these energy-rich substrates are not able to prevent the impairment of cognitive performance during acute hypoglycemia. The reason could be that burning lactate and/or fatty acids does not release redox energy sufficiently for oxidative phosphorylation to compensate for the decreased ATP generation during acute hypoglycemia. In contrast, there is evidence that neuronal firing is supported by glucose as the main fuel. That would enable the rapid supply of ATP to neuronal terminals through non-oxidative glucose degradation.

## 1. Introduction

The brain’s energy needs in humans are impressively illustrated by the fact that an organ amounting to only 2% of the whole-body mass consumes 20% of the total oxygen intake at rest [1]. The brain is also never completely at rest, since there are always firing neurons. Neurons contribute up to 80% to the cerebral energy turnover [2]. However, despite the brain’s high energy requirement, it does not have significant stores of glucose or of alternative energy-rich fuels. Moreover, a lack of glucose in the human body is mostly caused by insulin-induced acute hypoglycemia in diabetics, by the decoupling of neonates from the maternal bloodstream at birth, or by prolonged starvation. In order to maintain cerebral energy metabolism during prolonged starvation, the liver supplies the brain via the circulation with glucose synthesized from carbon skeletons of amino acids derived from muscle protein. In addition, it provides ketone bodies (KBs, β-hydroxybutyrate plus acetoacetate) derived from the β-oxidation of fatty acids (FAO) [3]. Thanks to this hepatic fuel supply, Stone Age people and their successors survived during periods of long-term food shortage in the past. However, during an insulin-induced acute glucose shortage, the hepatic fuel supply is suppressed, causing an “energy crisis” in the brain due to the shortage of sufficient ATP to support its activities, such as the transmission of multiple signals. Acute hypoglycemia can also occur when marathon runners suffer from extreme physical exhaustion, or when diabetics are exposed to medical treatment with glucose-lowering sulfonylureas. The lack of energy in the brain becomes visible as disorientation (marathon runners) or falling into unconsciousness (diabetics). Nevertheless, the energy crisis associated with acute hypoglycemia remains somewhat surprising, because the circulation can in principle provide the brain with lactate and fatty acids as other energy-rich fuels.

In this short contemplation, the very basics of human brain energy metabolism are reviewed first. Thereafter, similarities and differences between the above-mentioned hypoglycemic situations are discussed. Finally, a state-of-the-art overview of the suitability of lactate or fatty acids as energy-rich fuels during acute, severe hypoglycemia to protect the brain’s activities is given.

## 2. Brain Energy Metabolism at a Glance

The brain’s high energy demand arises from a plethora of ATP-consuming reactions, most notably the restoration of ion gradients across neuronal membranes, synaptic transmission, the axonal and dendritic transport of proteins or organelles by kinesin and dynein motor proteins, and the reuptake and regeneration of neurotransmitters. Synaptic signaling accounts for the largest portion of total cerebral energy consumption [4]. It should be noted that the brain’s energy metabolism is complex and compartmentalized, as uncovered by comparing white and gray matter, or neurons and glia cells with each other [1]. Even neurons have varying energy requirements, because excitatory neurons consume more energy than inhibitory neurons [5]. Notably, synaptic mitochondria differ from the remaining brain mitochondria (often termed non-synaptic mitochondria) in characteristics such as a smaller size and a different functional fingerprint [6,7].

Traditionally, glycolytic degradation of glucose combined with the oxidation of pyruvate inside the mitochondria is considered the most important source of reducing equivalents (NADH, FADH_2_) for supporting oxidative phosphorylation with redox energy [8]. Glucose crosses the blood–brain barrier (BBB) with the help of the glucose transporter GLUT-1, and subsequently, glucose is taken up by neurons with the assistance of GLUT-3 and GLUT-4 [9]. Neurons and astrocytes apply different strategies for ATP regeneration with glucose as fuel [10]. Astrocytes have a constitutively high glycolytic capacity, being equipped with a robust activity of the 6-phosphofructo-2-kinase/fructose-2,6-bisphosphatase-3 (PFKFB3), and a pyruvate dehydrogenase complex (PDC) that is constantly inhibited by phosphorylation [11]. Therefore, astrocytes, as the main consumers of glucose in the brain, regenerate ATP predominantly by glycolysis. In contrast, due to the continuous elimination of the bifunctional PFKFB3 enzyme via proteasomal degradation, neurons have a lower glycolytic capacity but a maximally active PDC, features that enable neurons to regenerate ATP mostly by oxidative phosphorylation.

Assuming that ATP regeneration from ADP occurs exclusively by oxidative phosphorylation with glucose as the sole fuel, the oxygen-to-glucose stoichiometry should theoretically be 6:1. However, calculated ratios of CMRO_2_/CMRglucose from measured central metabolic rates of oxygen (CMRO_2_) and of glucose (CMRglucose) were reported to be lower [12]. This gave rise to the assumption that glucose is converted non-oxidatively to lactate (also known as aerobic glycolysis) and/or that the consumption of fuels other than glucose contributes to ATP regeneration [13,14]. Lactate is the thermodynamic end-product of the glycolytic degradation of glucose [15], and it is formed under anaerobic as well as fully aerobic conditions in tissues at physiological concentrations of 0.5 to 20 mM [16]. Lactate is considered an energy-rich substrate of tissue energy metabolism, including that of the human brain [10,16,17]. There is also evidence that lactate has the potential to promote FAO via the tricarboxylic acid (TCA) cycle [18].

Discussion regarding FAO, and particularly its extent in brain tissue, dates back half a century, and it is still ongoing [19,20,21,22,23,24,25,26]. Until the end of the last century, it was believed that FAO contributed only to a negligible extent to energy homeostasis in the brain. Thereafter, interest in FAO in the brain was rekindled, and with new experimental approaches, the capacity of FAO was substantiated [22,23,27]. Evidence for FAO in brain tissue was obtained with a mouse model lacking carnitine palmitoyltransferase 2, a component of the carnitine shuttle system for the entry of long-chain fatty acids into the mitochondria [23]. The KO mice are unable to oxidize long-chain fatty acids, and consequently acyl carnitines accumulate in brain tissue. In addition, perfusion of the rat brain with 13C-labeled octanoate (caprylic acid; C8:0), and the subsequent analysis of its metabolism by means of NMR spectroscopy have uncovered that oxidation of octanoic acid could amount to up to 20% of the total energy demand of the brain [27]. Notably, FAO is strongly compartmentalized in the brain and is predominantly involved in the energy metabolism of astrocytes [23,26]. However, evidence for FAO in neurons, and also in the synaptic terminals, was recently found in sophisticated experimental studies [28,29]. This was shown by using genetic ablation of a neuron-specific triglyceride and phospholipid lipase, DDHD2 [28]. The resulting deficiency in long-chain saturated free fatty acids for FAO impairs mitochondrial respiration and ATP synthesis in cultured neurons. Notably, the DDHD2 lipase supplies mitochondria of synaptic terminals with fatty acids derived from lipid droplets [29]. Regardless of this new, surprising insight into neuronal bioenergetics, FAO takes place mostly in astrocytes. This fact leads to the question: why do astrocytes have a moderate capacity for FAO, when these cells have otherwise high glycolytic activity to sustain their relatively low energy demand? First, acetyl-CoA generated by astrocytic FAO is predominantly channeled into ketogenesis rather than fueling the TCA cycle [30]. Astrocytes possess the enzymatic equipment for the synthesis of KBs, which they make available as fuel to neurons [31,32]. In addition, astrocytes utilize acetyl-CoA for synthesizing cholesterol, which is a vital component for synapse formation and membrane fluidity [33].

Moreover, astrocytes, as an interface between blood vessels and neurons, play a crucial role in fuel supply from the circulation to neurons [10,34,35,36]. Apart from KBs, astrocytes supply glucose and lactate as fuels to neurons [10,26,37]. There is also reason to speculate that glucose saved by the oxidation of KBs is used in the pentose-phosphate pathway for NADPH generation, thereby improving the neuronal antioxidative defense [21]. Besides being a fuel supplier, astrocytes coordinate pathways that modulate neuro-glia communication and cognitive outcomes using lactate and reactive oxygen species (ROS) as signal molecules [26,30,38]. Notably, enhanced astrocytic ROS generation results from the joint activities of FAO and a loosely assembled mitochondrial respiratory chain [30].

## 3. Starvation-Induced Hypoglycemia and Counter-Regulation

**Figure 1:**
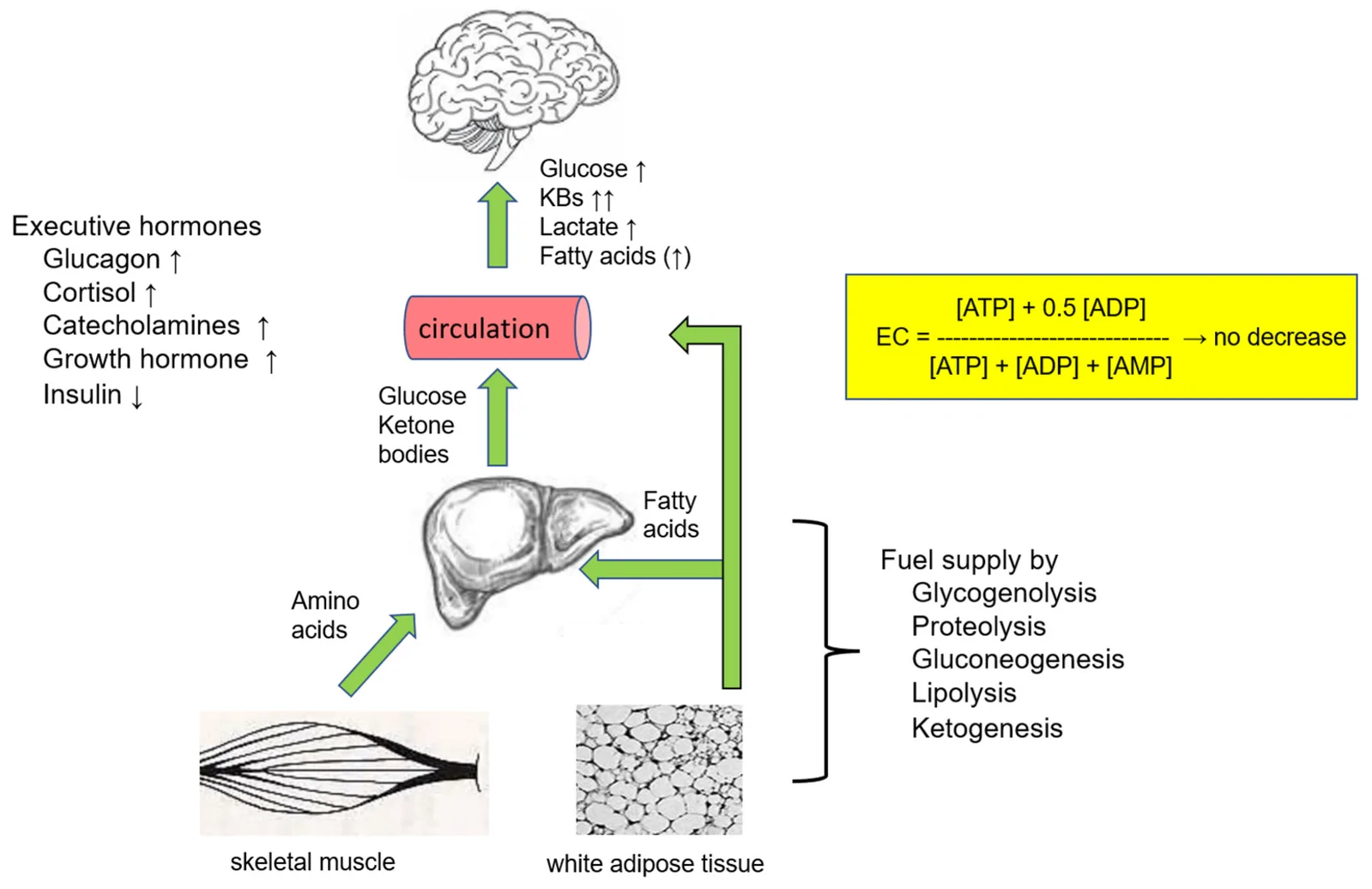
Supply of energy-rich substrates to the brain during long-term starvation. The liver supplies the brain via the circulation with glucose and ketone bodies (KBs) (β-hydroxybutyrate plus acetoacetate) during long-term starvation. Low levels of insulin in the bloodstream promote gluconeogenesis and ketogenesis. Cortisol and catecholamines trigger the proteolytic degradation of skeletal muscle protein, thereby liberating amino acids with carbon skeletons for gluconeogenesis. Catecholamines and glucagon directly or indirectly activate the hormone-sensitive lipase in white adipose tissue. β-Oxidation of fatty acids generates acetyl-CoA, which is utilized as a substrate for the synthesis of KBs. KBs oxidation can provide up to 50% of the brain’s total energy needs [47]. Growth hormone secretion opposes insulin effects on glucose metabolism. Notably, even during prolonged starvation, the glucose concentration in the bloodstream does not drop below 2.8 mM, and the energy charge (EC) in brain tissue is barely reduced. The EC is a measure of a cell’s energy status, reflecting the balance of ATP, ADP, and AMP, typically ranging from 0.8 to 0.95 in healthy cells [48]. The concept of EC was introduced to indicate how “full” a cell’s energy battery is at any given moment.

Long-term starvation lowers the availability of glucose for the energy metabolism of organs and causes hypoglycemia. Recovery from hypoglycemia is termed counter-regulation and involves the suppression of endogenous insulin secretion and the activation of hormone secretions, such as glucagon, adrenaline, and others [39]. Glucose-sensing neurons in the hypothalamus and in other brain regions respond first to decreased blood glucose levels, which are normally maintained in a range from 4 to 8 mmol/L [40]. In addition, the cerebral glucose concentration is linearly related to that in the blood, but about 3- to 5-fold lower [41]. There are also glucose-sensing cells in various regions of the body, such as the pancreas, mouth, and gastrointestinal tract. All of these cells are connected to a systemic network, including the cells that release executive molecules such as insulin, glucagon, catecholamines, cortisol and growth hormone. Consequently, this network triggers the activation of glycogenolysis, gluconeogenesis, and ketogenesis to supply cells with glucose and KBs via the circulation [3]. As a first response to an unphysiologically low blood glucose concentration, insulin secretion is halted via distinct neuronal innervation of pancreatic tissue by both sympathetic and parasympathetic nerve fibers [42]. Consequently, glucose uptake by insulin-sensitive organs (liver, muscle, and white adipose tissue) is blocked, and in addition, the insulin-dependent inhibition of glycogenolysis and gluconeogenesis is removed [43]. It is also known that growth hormone opposes insulin activities in glucose metabolism [44]. Moreover, glucagon and catecholamines (epinephrine, and norepinephrine) trigger the release of glucose from glycogen in the liver and skeletal muscle by the activation of glycogen phosphorylase. By these activities, the glucose level in the blood is generally maintained at about 2.8 mM, even under the most extreme starvation situations. Catecholamines also contribute to the enhanced secretion of glucagon by the activation of β_2_-adrenergic receptors on pancreatic α-cells. Glucagon stimulates gluconeogenesis, and otherwise, inhibits hepatic glucose uptake. Furthermore, catecholamines activate lipolysis in white adipose tissue, a process that elevates the levels of non-esterified fatty acids (NEFAs) in the circulation, and thereby, provides peripheral tissues with NEFAs as an alternative fuel [40,45]. With enhanced FAO in the liver, ketogenesis is fueled with acetyl-CoA for the generation of KBs, which are transported via the circulation to the brain. After crossing the BBB, KBs are converted to acetyl-CoA, which is now accessible for oxidation by the TCA in neurons and glial cells. NEFAs support gluconeogenesis in the liver with energy released by FAO, and simultaneously reduce glucose degradation in muscle. Cortisol activates hepatic gluconeogenesis and ketogenesis, and also lipolysis in fat depots [46]. Together, by the hepatic release of glucose and KBs into the circulation, the brain’s energy homeostasis is maintained, and thereby the brain is protected against the impairment of cognitive functions for a period of several weeks (Figure 1).

## 4. Neonate Hypoglycemia

During pregnancy fetuses receive a continuous supply of glucose from the maternal circulation [49], however, birth interrupts the maternal supply of glucose to the newborn. Moreover, the newborn brain has a high energy need, which accounts for up to 60% of the total body oxygen consumption while representing only 13% of the body weight [50]. Sudden postnatal hypoglycemia can be a severe complication of the cerebral energy metabolism of preterm newborns due to their shortage of stores of high-energy fuels [51]. However, birth also initiates the immediate secretion of glucagon, catecholamines and cortisol into the neonatal circulation [52]. Secretion of these executive molecules is also indirectly stimulated by a low plasma insulin level. Glucagon activates hepatic glycogenolysis, which rapidly increases systemic glucose through the degradation of stored glycogen. In the early neonatal period, an increase in blood glucose levels is indirectly promoted by the activation of lipolysis, FAO, and proteolysis. In addition, hepatic ketogenesis becomes increasingly active as a source to supply the neonatal brain with KBs as alternative fuels. Strikingly, the uptake of KBs by the neonatal brain is up to five times faster than that in adults [50]. Because of the low enzymatic capacity of gluconeogenesis in the early neonatal phase, this pathway contributes to plasma glucose homeostasis only a few hours later. Together, from birth to the onset of breastfeeding, an energy crisis in the newborn brain seems to be prevented in a manner similar to prolonged starvation. At the same time, the enzymatic capacity of oxidative phosphorylation in the brain mitochondria of neonates matures rapidly, as documented by insights into the perinatal development of the rat brain [53].

## 5. Acute Hypoglycemia

**Figure 2:**
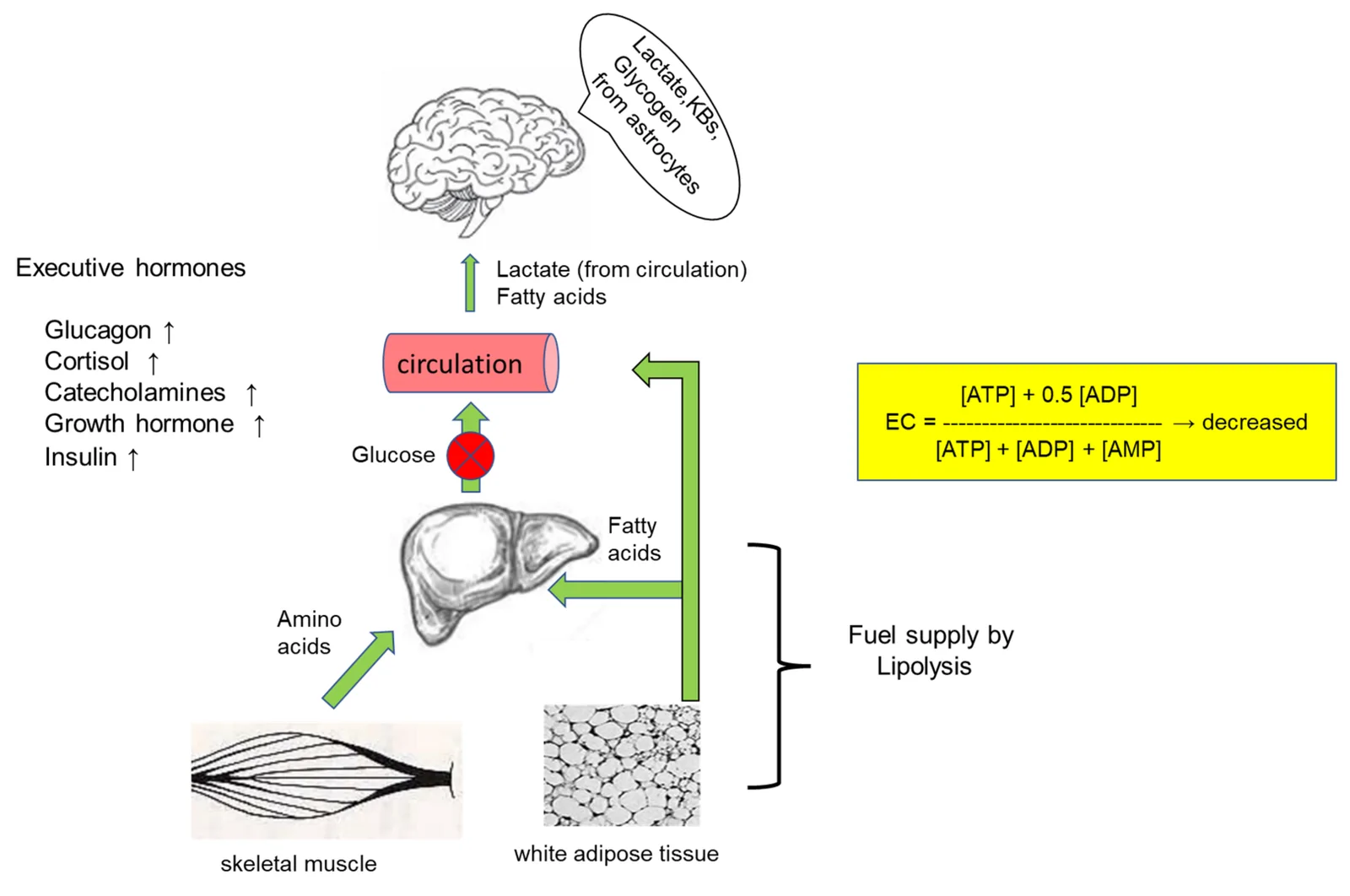
Availability of energy-rich substrates in the brain during acute hypoglycemia. In acute (insulin-induced) hypoglycemia, the hepatic blockade of glycogenolysis and gluconeogenesis by high insulin levels prevents the liver from donating glucose to the brain. Compared to the liver or musculature, the glycogen content in the brain is significantly lower (~5 µmol per g of brain tissue [57]). Nevertheless, it has been postulated that brain glycogen serves as an energy store by supplying glucose and lactate to neurons during hypoglycemia [58]. Astrocytes extract fatty acids from the circulation for β-oxidation and generate KBs, which are donated to neurons.

Acute hypoglycemia occurs frequently after the medical treatment of patients with diabetes using insulin or sulfonylureas. Curiously, short-term insulin-induced hypoglycemia was applied in the 1930s as a therapy to treat schizophrenia and other psychotic disorders [54], but this treatment was soon abandoned when the negative consequences of prolonged treatment became visible in patients. In insulin-induced (iatrogenic) hypoglycemia in patients with diabetes, apart from the low concentration of blood glucose due to insulin-driven uptake of glucose in skeletal muscle and adipose tissue, insulin blocks the release of glucagon, thereby inhibiting glycogenolysis and gluconeogenesis in the liver. In contrast, lipolysis becomes rapidly activated, thereby increasing the level of fatty acids in the circulation [55], and surprisingly, the inhibitory effect of insulin on hepatic ketogenesis is overcome by the release of counterregulatory hormones [56]. The fuel supply to the brain during acute hypoglycemia is summarized in Figure 2.

Moderate hypoglycemia (blood glucose level ranging from 2.2 to 3.3 mM) affects EEG activity and cognitive functions [59], whereas severe hypoglycemia (blood glucose level is below 1 mM) has the potential to kill vulnerable neurons, most likely those in the cerebral cortex and the hippocampus [60]. 31P-NMR spectroscopy uncovered that in severe hypoglycemia, the energy status of the brain becomes markedly lowered, and a positive correlation between the cerebral phosphocreatine/phosphate signal and the blood glucose level was found [61]. This finding demonstrates that cerebral energy-dependent functions are more or less impaired by low concentrations of glucose. About half a century ago, it was already reported that ATP depletion in rat brain tissue is paralleled by the onset of electrocerebral inactivity (referred to as a flat-line in EEG), and astrocytic glycogen depletion [62]. Furthermore, a decreased energy charge (EC) in hypoglycemic brain tissue triggers an elevation in the level of NEFAs by membrane phospholipid hydrolysis [63]. Taking into account the lipotoxic potential of long-chain NEFAs [21,64], it is likely that enhanced tissue levels of these fatty acids contribute to cell damage during acute severe hypoglycemic episodes.

However, the hypoglycemic death of neurons does not follow directly from a glucose shortage. Instead, the death of neurons results from a process consisting of multiple events [65]. First, neuronal depolarization initiates the presynaptic release of glutamate, aspartate, and zinc (Zn^2+^). Impaired glutamate uptake by astrocytes consequently causes a highly elevated extracellular level of glutamate. Both glutamate and aspartate are known to activate glutamate receptors, thereby promoting excitotoxicity [66]. In addition, zinc influx into the synaptic cleft stimulates ROS generation from mitochondria or NADPH oxidase. Increased ROS-induced DNA damage activates poly (ADP-ribose) polymerase-1, which is a consumer of cytosolic NAD^+^. Due to this NAD^+^ deficiency, cells become unable to use glycolysis for energy gain. Finally, these events make cells prone to death via mitochondrial permeability transition [67].

In addition, at first glance, insulin-triggered acute hypoglycemia resembles the sudden separation of the newborn from the maternal circulation at birth. However, the well-filled energy stores of glycogen and triglycerides in full-term newborns prevent an energy crisis in their brains up to the onset of breastfeeding. In addition, contrary to the insulin-induced glucose shortage in neonatal hypoglycemia, insulin does not suppress hepatic ketogenesis, and thereby the delivery of KBs to the brain is maintained.

## 6. Use of Lactate and Fatty Acids as Fuels During Acute Hypoglycemia

### 6.1. Lactate

Oxygen consumption of the human brain remains constant after switching from euglycemia to moderate hypoglycemia [68]. This surprising finding was interpreted as suggesting that alternative energy-rich substrates are oxidized to compensate for a decrease in ATP generation during glucose deficiency in the hypoglycemic brain. The most likely substitutes for glucose are lactate and fatty acids in the circulation. Systemic lactate concentration is known to increase during insulin-induced hypoglycemia [69]. Lactate is transported from the bloodstream into the brain parenchyma by facilitated transport via the nonspecific proton-monocarboxylate symporter 1 (MCT1) and by diffusion. Lactate is taken up and released by MCT1 of astrocytes and oligodendrocytes. Reported values of Km and Vmax for astroglia cells are 7.7 mM and 250 nmol/(min x mg of protein) [70]. In contrast, lactate released from glia cells is taken up by neurons using the high-affinity transporter MCT2 (Km 0.74 mM) [71,72].

**Figure 3:**
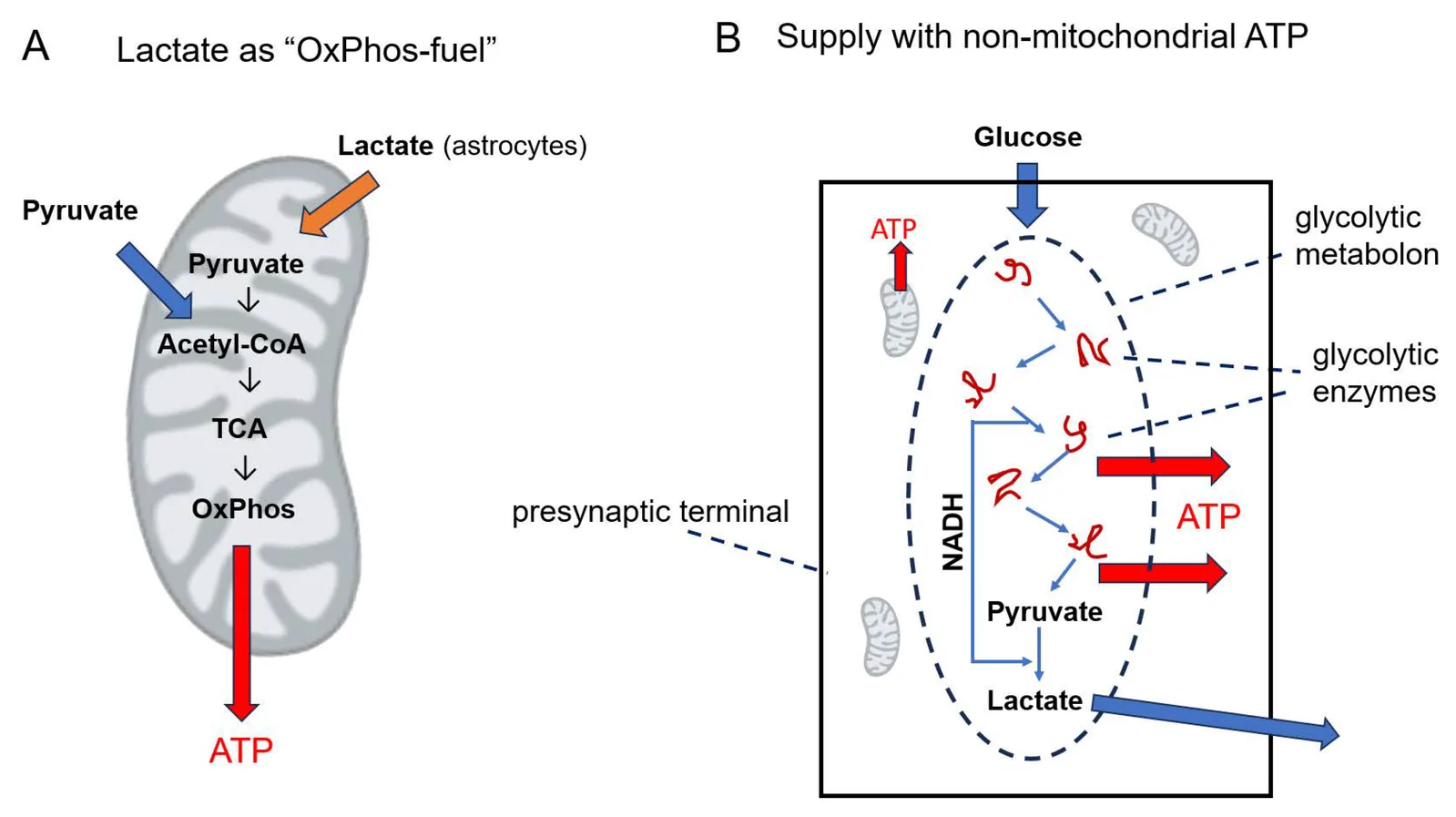
Supply of firing neurons with lactate as “OxPhos-fuel” or with non-mitochondrial ATP regenerated by glycolytic degradation of glucose. (**A**) Supply of lactate to mitochondria as energy-rich fuel. In addition to pyruvate, the oxidation of lactate, released from activated neighboring astrocytes, enables mitochondria in nerve terminals to enhance their ATP output. (**B**) Rapid ATP supply by non-oxidative degradation of glucose (aerobic glycolysis). Synaptic transmission triggers the assembly of glycolytic enzymes into a metabolon in nerve terminals. This reorganization of cytosolic enzymes enables sustained synaptic transmission by quick glycolytic ATP regeneration.

Early evidence for considering lactate as an energy-rich fuel in cerebral energy metabolism was obtained from experimental work on rat hippocampal slices, substantiating that lactate as a sole energy source is able to sustain normal synaptic functions [13]. This finding matches the high activity of LDH-1 in the synaptic compartment, an isoenzyme favoring the formation of pyruvate from lactate [73]. Support of synaptic energy-dependent activities by lactate was not impaired by the glycolysis inhibitor iodoacetic acid, indicating that lactate is oxidized after conversion to pyruvate by the combined activities of pyruvate dehydrogenase and the TCA cycle (Figure 3). The astrocyte-neuron lactate shuttle hypothesis (ANLS) postulates that neurons receive energy support through lactate oxidation [74]. In line with this view, firing glutamatergic neurons receive lactate from astrocytes. This support is stimulated by the ATP-consuming Na^+^-driven reuptake of glutamate from the synaptic cleft. The strong belief in lactate as an important cerebral fuel has even raised the question [75]: is lactate used as the ultimate cerebral oxidative energy substrate?

However, there are arguments against the direct mitochondrial oxidation of lactate to pyruvate [76]. According to this view, the high enzymatic activity of the near-equilibrium enzyme LDH combined with the highly reduced matrix (NADH plus NAD^+^) pool does not support lactate oxidation inside mitochondria. This view is supported by in silico modeling, proposing that matrix-LDH activity would prevent the oxidation of pyruvate when it is added to incubations of isolated mitochondria [76]. On the other hand, there is evidence that mitochondria take up lactate from the environment and subsequently oxidize it in the matrix compartment [77]. However, the general assumption is that glycolytically generated pyruvate is taken up from the cytosolic cell compartment, and after its transfer through the inner mitochondrial membrane, it is oxidized by pyruvate dehydrogenase to acetyl-CoA and NADH in the matrix. Nevertheless, this common textbook view leads to the question: In what way is the glycolytic intermediate pyruvate diverted to the mitochondria, because the high activity of cytosolic LDH as well as thermodynamics suggest its rapid reduction to lactate? In response to this problem, a mitochondrial lactate oxidation complex (mLOC) was proposed, consisting of assembled LDH (attached to the cytosolic side of the inner mitochondrial membrane), complex IV of the respiratory chain, a monocarboxylate transporter (MCT1) and the chaperone protein CD147 [16]. This proposal states that pyruvate is generated from lactate by oxidation at the surface of the inner mitochondrial membrane, and after transport through the inner membrane, pyruvate is oxidized inside the mitochondrial matrix.

Besides the pros and cons of lactate oxidation in mitochondria, what can experimental work tell us about the use of lactate as fuel in mammalian brain energy metabolism during hypoglycemia? First, an increase in the systemic concentration of lactate as well as the membrane content of lactate transporters was reported during hypoglycemia [69,78]. Normal human volunteers were put into a stable state of moderate hypoglycemia (3 mM blood glucose) [68]. Both glucose and lactate uptake by the brain were estimated at euglycemia and hypoglycemia as a product of cerebral blood flow (CBF) and the arteriovenous difference (A-V)_diff_. Loss and gain of energy by changes in the brain fuel uptake revealed that not more than 25% of hypoglycemia-induced loss of ATP generation was compensated by lactate. This result is in agreement with a study done with healthy individuals exposed to an intravenous lactate infusion [17]. At rest, cerebral lactate utilization changed from a net lactate release (of 0.06 ± 0.01 mmol/min) to an uptake (of 0.15 ± 0.07 mmol/min) during lactate infusion. Furthermore, the uptake of lactate was paralleled by a decrease in glucose uptake, and systemic lactate contributes to cerebral energy expenditure by no more than 27 ± 4%.

To examine a possible ability of lactate to restore brain activity during hypoglycemia, rats were first exposed to a threefold recurrent hypoglycemia during a 3-day period, and thereafter infused with lactate [35]. Adaptation of the rat metabolism to recurrent hypoglycemia results in a twofold increase in the lactate uptake from the blood to the brain; however, lactate uptake was paralleled by a glucose uptake from the circulation. This surprising finding has been explained by a lactate-stimulated glucose uptake at hypoglycemia from blood into the brain. Based on this observation, it was speculated that lactate acts more likely as a “metabolic regulator” of the glucose supply to the brain, but not as a fuel itself [35].

In summary, lactate as a substrate supports the energy metabolism of the brain insufficiently, and consequently, lactate can only partly replace glucose as a fuel in acute severe hypoglycemia [17,68,79]. Moreover, administration of lactate without glucose is not sufficient to promote EEG recovery from an isoelectric state during hypoglycemia [80]. Strikingly, from a study with hippocampal slice cultures it was reported that at physiological concentrations of lactate and glucose, glucose is the preferred fuel, but at elevated pathophysiological concentrations, lactate is preferred [81].

### 6.2. Fatty Acids

Degradation of fatty acids by astrocytic FAO has been reported to be crucial for the protection of the brain’s functional integrity against neuroinflammation and neurodegeneration [24,82]. Furthermore, astrocytes eliminate damaged fatty acids in neurons, which are formed by attacks of ROS on membrane phospholipids during firing episodes [83]. With mouse hippocampal slices, it has been found that after a traumatic brain injury, the oxidation of octanoate can partially restore hippocampal bioenergetics [84]. This finding raises the question: could FAO improve cerebral energy metabolism during acute hypoglycemia? So far, only a few research studies have addressed this issue. In one study, type 1 diabetic and nondiabetic humans were infused with labeled acetate during hypoglycemia (blood glucose level was lowered to 3 mM via insulin infusion), and the uptake as well as the oxidation of acetate in the brain was studied using NMR spectroscopy [85]. Generally, acetate is transported from the circulation into astrocytes, and from there to neurons by the monocarboxylate transporters (MCT1 and MCT2). A twofold higher acetate level in the brain tissue of diabetic humans was found, compared with that of nondiabetics, and this was paralleled by a twofold increase in the rate of cerebral acetate oxidation in the diabetics. Nevertheless, because of the low protein level of acetyl-CoA synthetase 2 and the relatively high enzymatic activity for the hydrolysis of acetyl-CoA, acetate is a poor substrate, especially in neurons [86].

Moreover, patients with type 1 diabetes were examined to see whether an ingestion of triglycerides of medium-chain fatty acids improves their cognitive performance during hypoglycemia [87]. Before discussing the obtained results, it might be of interest to mention some characteristics of medium-chain fatty acids (MCFAs), such as octanoate. Contrary to long-chain fatty acids (LCFAs), MCFAs are transported in the bloodstream without a protein carrier. MCFAs are taken up carnitine-independently by mitochondria, and are activated intramitochondrially to acyl-CoA thioesters (see [88] for a review). Compared with LCFAs, MCFAs exert only weak protonophoric and lytic activities in mitochondria and do not significantly impair electron transport in the respiratory chain. As an energy source for tissue metabolism, triglycerides of MCFAs have advantages compared to those of LCFAs [88]. They are more rapidly digested in the gut, do not become incorporated into chylomicrons and their tissue metabolism does not depend on proteins for binding and transport. MCFAs are transported by the portal bloodstream to the liver, where they are readily metabolized.

In the study [87], it was also measured by incubating rat hippocampal slices with a low glucose concentration (2 mM) to determine whether an addition of β-hydroxybutyrate or octanoate affects synaptic transmission. Oral application of triglyceride-bound octanoate to hypoglycemic diabetics partially restored their cognitive performance. However, in contrast to the ketone body β-hydroxybutyrate, the MCFA octanoate failed to support synaptic transmission of hippocampal slices at low glucose concentrations.

Moreover, synaptic transmission of hippocampal slices ceased completely in the absence of glucose, and notably, β-hydroxybutyrate was not able to improve synaptic transmission. These findings might be explained as follows: ingestion of triglycerides of MCFAs by diabetics stimulates the generation of KBs by hepatic ketogenesis, which benefits neuronal performance. Otherwise, a lack of effect of octanoate on the synaptic transmission of hippocampal slices at a low glucose concentration indicates that octanoate oxidation is not sufficient to partially substitute for glucose as an energy-delivering substrate, contrary to β-hydroxybutyrate. However, it should also be added that octanoate is able to improve cerebral energy metabolism directly by supplying the TCA cycle with acetyl-CoA generated from its β-oxidation. From a study using hippocampal slices from mice, it was reported that octanoate helps to restore mitochondrial oxidative phosphorylation after traumatic brain injury [84]. This finding also leads to the question of whether the ATP regeneration with fatty acids as an energy source contributes to the energy homeostasis of neurons. In this context, it is important to remember the reported evidence of FAO in neurons, and also in the synaptic terminals [28,29]. Nevertheless, taken together, the rate of ATP regeneration based on the overall process of the utilization of fatty acids supplied from the bloodstream is insufficient to substitute for glucose in rapid ATP delivery. Possible explanations for this deficiency were discussed previously [21].

## 7. Concluding Remarks

Diabetics can fall into a life-threatening state of unconsciousness after medication with insulin or sulfonylureas due to the onset of acute hypoglycemia. The relatively high frequency of this medical emergency reflects the brain’s vulnerability to a rapid shortage in blood glucose levels. The human liver (wet-weight 1.5 to 2 kg) stores about 7% glycogen [89], equivalent to about 135 g of glucose. When comparing the amount of hepatic glucose with the brain’s need for about 140 g of glucose per day, a partial disruption of cognitive functions by an energy shortage shortly after a medical treatment should not happen. Instead, during acute hypoglycemia triggered by insulin treatment, hepatic glucose is not released into the circulation due to the insulin-dependent blockade of glycogenolysis. Moreover, it is not likely that brain fueling by the combined mitochondrial oxidation of lactate and fatty acids supports oxidative phosphorylation with sufficient redox energy for the compensation of decreased ATP generation during acute hypoglycemia [17,19,23,27,68,80].

Furthermore, several reports document that FAO takes place predominantly in astrocytes [23,26,31] and that high-energy-demanding neurons fight against FAO because of enhanced ROS formation and poor neuronal antioxidative defense [21,83,90]. In addition, neurons are equipped with high acyl-CoA thioesterase activity to limit FAO [91]. Aside from these facts, fatty acids are not always completely oxidized by astrocytes, because acetyl-CoA is also used for ketogenesis, de novo synthesis of brain lipids, and the anaplerotic generation of TCA cycle intermediates [26].

The brain’s cognitive function depends on multiple high-energy-dependent synaptic signal transmissions, and for that, nerve terminals have a large need for ATP [4,7]. Consequently, insufficient ATP availability or too slow availability of ATP for synaptic signal transmission is likely to impair cognitive neuronal functions. Moreover, stimulation of specific activities in local brain regions increased the local blood flow, a finding that indicates an increased local glucose uptake [92]. This finding has been explained by the firing of action potentials by neurons, increasing several times the energy need, mainly due to energy demands associated with synaptic transmission [93]. It has been discussed that sustained synaptic signal transmission by neurons requires an “extra-fuel support” for neurons by neighboring astrocytes, or an enhanced activity-driven glycolysis. There are good arguments that the rate of ATP regeneration by non-oxidative glucose degradation (aerobic glycolysis) is quicker than that by oxidative phosphorylation [94,95]. (1) Compared with the glucose uptake of activated cerebral regions, the oxygen uptake from the blood is less than demanded for complete combustion of absorbed glucose [14,92]. (2) Tissues that consume ATP at higher rates, such as the brain, rely heavily on glycolytic ATP production [96,97]. (3) Aerobic glycolysis increases locally with increased neuronal activity [98,99]. (4) Firing neurons do not rely on lactate; instead, they export lactate [100]. (5) Neuronal activation increases the cytosolic NADH/NAD^+^ ratio [101].

The hypothesis on ATP regeneration by non-oxidative glucose degradation with lactate as a glycolytic end product is further supported by the reorganization of glycolytic enzymes into a glycolytic metabolon (Figure 3), which accelerates glycolysis, and thereby ATP regeneration [99]. Thus, it has been reported that in the presynaptic terminals of the nematode Caenorhabditis elegans, glycolytic enzymes transiently cluster into a “glycolytic metabolon” at high energy demand [95], comparable to what is already found in fast-twitching fibers of exercising muscle [96]. It is also worth noting that ATP regeneration by non-oxidative glucose degradation would prevent the risk of impairing synaptic transmission by hypoxic events. Taken together, there is reason to hypothesize that the ATP supply during sustained synaptic signaling is driven by the combined ATP regeneration of non-oxidative glucose degradation plus oxidative phosphorylation. In addition, assuming this view, it is tempting to speculate that in firing neurons, fueled by ATP exclusively generated by mitochondria, oxidative phosphorylation would be the bottleneck.

The opinion presented here could also provide an answer to the question: Why are the most available energy-rich alternative substrates in the circulation, lactate and fatty acids, not able to protect the brain against an energy crisis during acute hypoglycemia? Despite the poor enzymatic capacity of FAO in brain tissue, ATP formation by mitochondrial oxidative phosphorylation is probably too slow to feed synaptic signaling processes with enough ATP alone. In addition, FAO is known to strongly stimulate the formation of ROS as toxic by-products of the respiratory chain reaction. Although the energetic efficacy of aerobic glycolysis for ATP regeneration is worse, this metabolic pathway regenerates ATP more rapidly than oxidative phosphorylation. Together, synaptic signaling driven by ATP regeneration based on aerobic glycolysis could explain that this process responds sensitively to a glucose shortage during acute hypoglycemia.

## Data Availability

No new data were created in this study. Data sharing is not applicable to this article.
